# Assignment of Vibrational Circular Dichroism Cross‐Referenced Electronic Circular Dichroism Spectra of Flexible Foldamer Building Blocks: Towards Assigning Pure Chiroptical Properties of Foldamers

**DOI:** 10.1002/chem.201903023

**Published:** 2019-10-23

**Authors:** Viktor Farkas, Adrienn Nagy, Dóra K. Menyhárd, András Perczel

**Affiliations:** ^1^ MTA-ELTE Protein Modelling Research Group Institute of Chemistry Eötvös Loránd University Pázmány P. stny. 1/A Budapest 1117 Hungary; ^2^ Laboratory of Structural Chemistry and Biology Institute of Chemistry Eötvös Loránd University Pázmány P. stny. 1/A Budapest 1117 Hungary

**Keywords:** amino acids, conformation analysis, circular dichroism, foldamers, secondary structure assignment

## Abstract

Assignment of the most established electronic circular dichroism (ECD) spectra of polypeptides and foldamers is either “evidence based” or relies on the 3D structures of longer oligomers of limited internal dynamics, which are derived from NMR spectroscopy (or X‐ray) data. Critics warn that the use of NMR spectroscopy and ECD side by side has severe limitations for flexible molecules because explicit knowledge of conformational ensembles is a challenge. Herein, an old–new method of comparing ab initio computed and measured vibrational circular dichroism (VCD) data is presented to validate both the structures (*conf*(*i*)) and their relative weights (*c*(*i*)) that make up the conformational ensemble. Based on the array of {*conf*(*i*), *c*(*i*)}, the pure ECD spectra, *g*(*i*)^conf(*i*)^, can be ab initio calculated. The reconstructed spectrum Σ*c*(*i*)*g*(*i*)^conf(*i*)^ can thus help to assign any experimental ECD counterparts. Herein, such a protocol is successfully applied to flexible foldamer building blocks of sugar β‐amino acid diamides. The epimeric pair of the model system was selected because these molecules were conformationally tunable by simple chemical modification, and thus, the robustness of the current approach could be probed. The initial hydrogen bond (NH⋅⋅⋅O) eliminated by N‐methylation reorients the amide plain, which influences the chiroptical properties of the foldamer building block; this structural change is successfully monitored by changes to the VCD and ECD transitions, which are now assigned to pure conformers. The current method seems to be general and effective without requiring extensive CPU and spectroscopic resources.

## Graphical abstract

**Secondary considerations**: Different amide plane orientations, coupled with spectral changes, that are modest or on large scale can be tracked by electronic circular dichroism spectroscopy (see figure). The method introduced herein allows complete secondary structure assignment of foldamers, chimera, and so forth composed of common (α‐) and/or non‐proteinogenic amino acid residues of a given length.

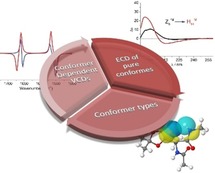

## Introduction

Both proteinogenic α‐ and non‐natural, but biocompatible, β‐, γ‐, δ‐, and *ϵ*‐amino acids are used as Lego‐like building blocks to modify and redesign proteins, chimera peptides, and foldamers.^1^, ^2^ To be able to tailor the structure and properties of these macromolecules, a firm understanding of the conformational properties of the building blocks themselves is required; thus, the structural elucidation of such molecules is a hot topic of current structural chemistry and biology. Both NMR and electronic circular dichroism (ECD) spectroscopic data, combined with molecular dynamics (MD) and applied quantum mechanical (QM) calculations play a significant role (in addition to other techniques (IR, Raman, etc.)) in deciphering their inherent structural properties. Although laborious NMR spectroscopy and/or X‐ray techniques are needed to determine high‐resolution structural data, chiroptical spectroscopy, both ECD and vibrational circular dichroism (VCD), is rapid in reporting global conformational features of the same macromolecules. For inherently dynamic systems, NMR spectroscopy provides time‐average restraints only, whereas X‐ray diffraction reports on the most stable conformer. Thus, VCD and ECD data, complemented by ab initio structures, are efficient and reliable complementary techniques, if used in combination. Herein, we report how VCD cross‐validated conformers, obtained at an appropriate level of theory, are especially useful to obtain and assign chiroptical properties, including ECD spectra of pure conformers of dynamic systems.

**Scheme 1:**
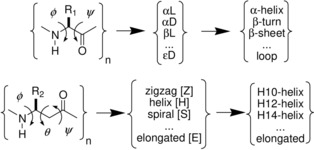
Two backbone dihedral angles (*φ*, *ψ*) characterize secondary structures of α residues, but three (*φ*, *θ*, *ψ*) of them per residue make up structures built from β residues.

Two dihedral angles per residue (*φ*, *ψ*) are sufficient to describe and characterize the major types of secondary structural motifs, such as α helices (α_L_, α_D_) and β sheets (β_L_), in polymers of α‐amino acid residues.^3^ However, for β residues, three backbone torsional angles (*φ*, *θ*, *ψ*) are required to provide a similar level of structural information and the basis of structural classification, such as zigzag (Z), helix (H]), spiral [S], and elongated (E) motifs (Scheme 1).^4^ However, the increase in complexity does not result in overall greater structural variability. β‐Amino acids have less flexible backbones^5^ because their sequentially adjacent amide bonds are separated more in space (insertion of an extra sp^3^‐C atom), which allows them to form locally more constrained, but also more stable, secondary structures.^6^

**Scheme 2:**
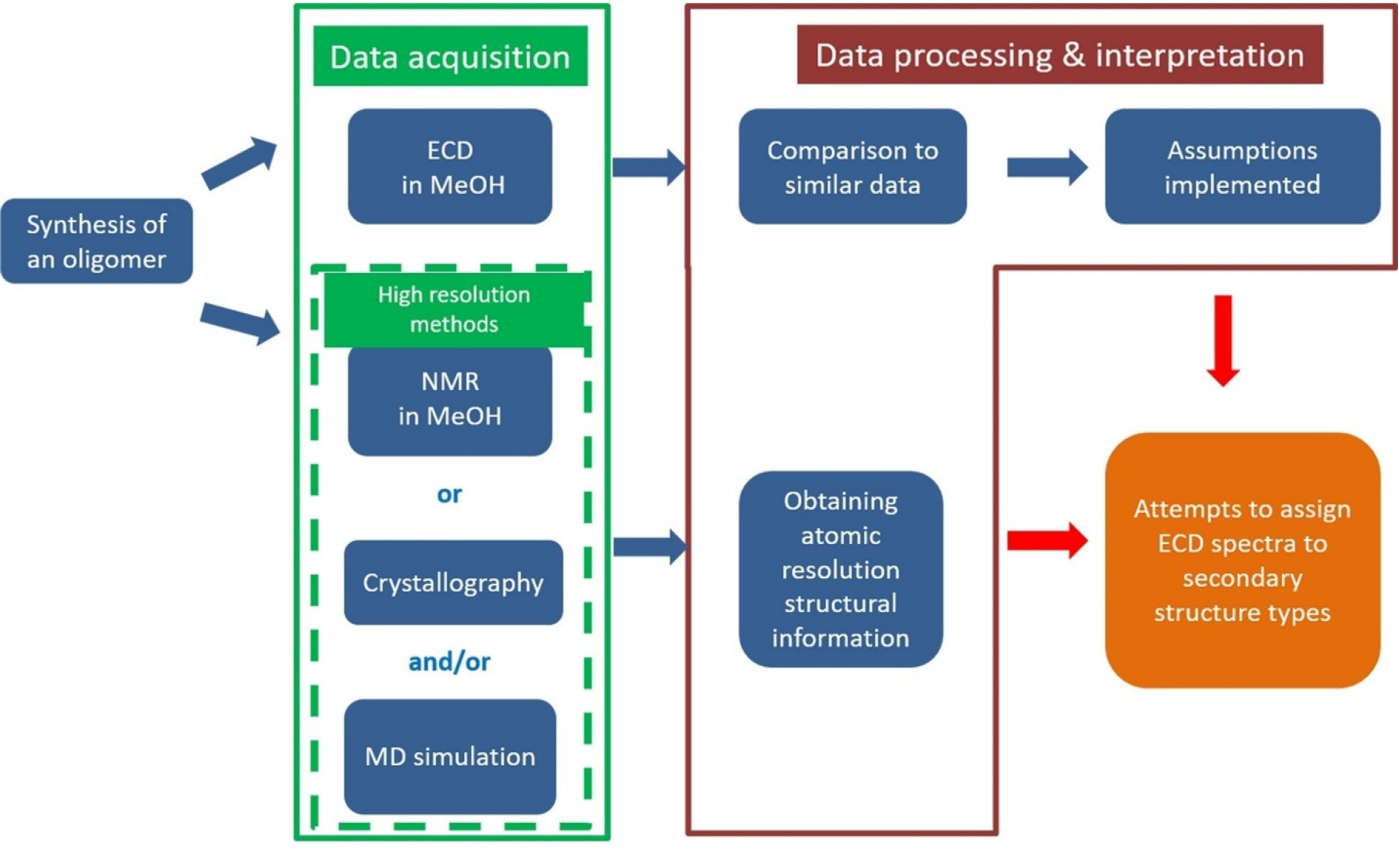
Attempts to assign ECD spectra of β peptides and foldamers.

ECD spectroscopy is a widely used chiroptical technique for the description of the conformational properties of polyamides in solution.^7^ Today, the ECD spectra of most secondary structural elements consisting of α‐amino acids are assigned and characterized: class A (β sheets), class B (II‐β turns), class C (α helix, I(III)‐β turns), class U (unordered or highly dynamic conformation), and so forth, following a seminal theoretical study by Woody and others.^8^, ^9^ Thus, ECD spectral patterns are assigned to characteristic backbone torsional subspaces: *φ*_A_±*ζ*, *ψ*_A_±*ζ*, *φ*_B_±*ζ*, *ψ*_B_±*ζ*, and so forth. For β peptides, however, a similar assignment library is not yet complete, in fact, it has hardly begun, although ECD spectra of dozens of polypeptides have been reported.^10^, ^11^ From the 1990s, with the appearance of foldamers, the field of non‐proteogenic amino acid containing polypeptides has expanded. Contrary to that of α peptides, for β peptides and foldamers, the structural assignment of ECD spectra is more challenging because there is a lack of meaningful spectra of evolutionary‐optimized typical protein folds, such as those used to decipher ECD properties of α‐amino acids in their oligomeric secondary structures. In the case of β peptides, the generalized scheme of data acquisition and interpretation (summarized in Scheme 2) starts with the synthesis of suitable β oligomers, followed by low‐ and high‐resolution data acquisition. The obtained high‐resolution structural information (NMR spectroscopy data and crystal structures) are directly assigned to the recorded chiroptical data (ECD, seldom VCD).

This method can be successfully applied to characterize ECD spectra of foldamers composed of β‐amino acids of limited backbone dynamics, for example, 2‐aminocyclopentanecarboxylic acid (ACPC for short). The groups of Gellman (*trans*‐*R*,*R*),^12^ Martinek (*cis*‐*R*,*S*),^13^ and Davies (*trans*‐*S*,*S*)^14^ used the hydrophobic β‐amino acid ACPC to make foldamers. They showed that, if the configuration of ACPC was set to *cis*, then the backbone fold of (ACPC)_*n*_ was exclusively extended. On the other hand, the homo‐oligomer built from *trans*‐ACPC adopted a helical overall fold. For the structural assignment of their ECD spectra, in line with Scheme 2, they used either crystal structures or NOE data.^12^, ^13^, ^14^ Thus, the ECD spectra measured in dilute solutions are directly attributed to structural information derived from 5–10 times more concentrated solution‐state data or from structural information determined by solid‐state characterization; this raised numerous concerns.^2^, ^15^, ^16^, ^17^ For example, Glättli et al. noted that, for H‐(β‐HVal‐β‐HAla‐β‐HLeu)_2_ and its dimethylated form (H‐[β‐HVal(Me_2_)‐β‐HAla(Me_2_)‐β‐HLeu‐(Me_2_)]_2_), which presented nearly identical ECD spectra, the NMR spectroscopy based structural assignment of a 3_14_‐helix was unlikely to be correct because the dimethylated variant could not form such a conformer due to steric repulsion.^15^, ^18^ In addition, the groups of Seebach and Gellman both published further examples, which illustrated that an NMR spectroscopy–ECD contradiction did exists for hybrid foldamers, specifically for systems composed of α‐ and β‐ or α‐ and γ‐amino acid residues.^2^, ^19^ The problem with the concept of using NMR spectroscopy as the high‐resolution referencing method for ECD arises from the fact that, for short and flexible oligomers, the available *J*‐coupling constants, NOE, and/or secondary chemical shift information is just too sparse for reconstructing ensembles of multiple backbone conformers. Furthermore, due to the timescale of NMR spectroscopy measurements, the acquired data reflect a time‐averaged 3D structure because the deconvolution of NMR spectra is not yet straightforward. In addition, crystallography provides a single (or few), rather than multiple, conformer, as expected for an inherently flexible oligopeptide. On the other hand, ECD spectroscopy, which is sensitive to small structural variations of the amide planes that are undetectable by NMR spectroscopy, is ideal for complementing or replacing these methodologies.^16^, ^17^

### Aims

Instead of using a single conformer determined by crystallography, or an NMR spectroscopy based condensed molecular structure, to attempt the assignment of ECD spectra to structures, our goal was to derive an alternative strategy. We propose herein to complete ECD assignment through cross‐validated VCD information, in combination with a structural ensemble derived from molecular mechanics (MM) and ab initio structure ensemble data and spectra. We demonstrate that, by using low‐resolution, absolute‐configuration‐controlled chiroptical methods, structural information of high significance can be gained, even in the case of flexible nanosystems.

We present herein an example how this old–new assignment strategy can be applied by using cyclic β‐amino acid derivatives of tunable backbone conformational properties. Our pair of aminofuranuronic acid (AFU) diamides (namely, the C‐3 epimeric pair of *N*,*N*‐dimethyl‐1,2‐*O*‐isopropylidene‐3‐azido‐3‐deoxy‐α‐d‐ribofuranuronamide (**1 a**) and *N*,*N*‐dimethyl‐1,2‐*O*‐isopropylidene‐3‐azido‐3‐deoxy‐α‐d‐xylofuranuronamide **2 a**)) and their *N*‐methylated variants (*N*,*N*‐dimethyl‐1,2‐*O*‐isopropylidene‐3‐amino‐3‐deoxy‐α‐d‐ribofuranuronamide (**1 b**) and *N*,*N*‐dimethyl‐1,2‐*O*‐isopropylidene‐3‐amino‐3‐deoxy‐α‐d‐xylofuranuronamide (**2 b**)) are good examples to show how a small change (H↔CH_3_) may affect the overall conformation and how such backbone conformational shifts can be effectively recorded and deciphered by chiroptical methods.

## Results and Discussion

**Scheme 3:**
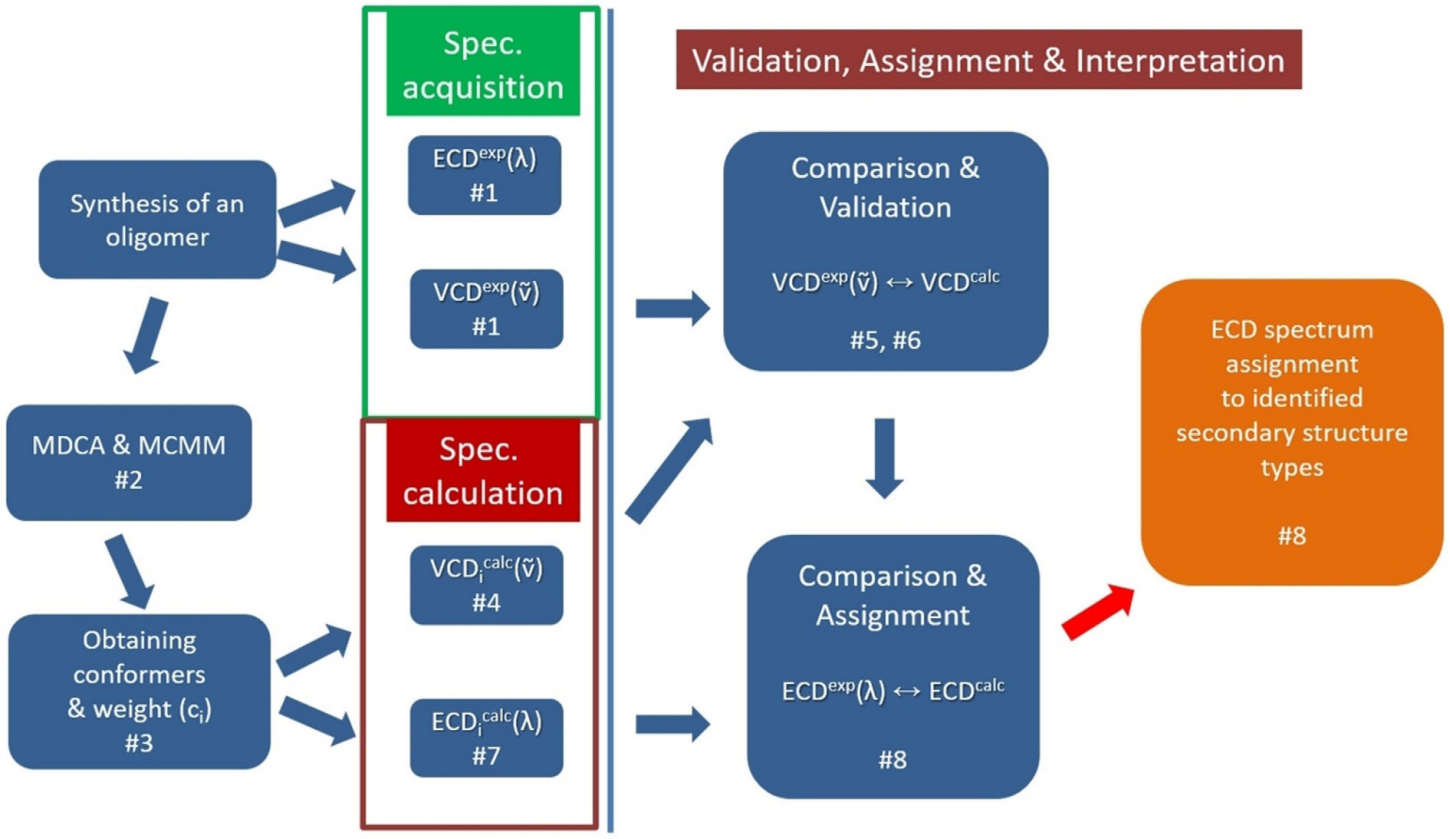
The new approach to assign ECD spectral properties to backbone structures of flexible oligo‐ and polypeptides, foldamers, and so forth.

The characterization of polypeptide building blocks by ECD could be a promising approach, as mentioned above; however, it would require spectral deconvolution and the assignment of pure ECD spectra.^19^, ^20^, ^21^ The relative orientation of chirally perturbed consecutive amide planes, in line with a report by Niggli et al.,^17^ fundamentally influences the shape of the ECD spectra. However, in the case of conformational ensembles, the determination of all backbone structures seems to be a key, but demanding, task (multidimensional conformation analysis (MDCA)).^21^, ^22^, ^23^ For α‐amino acid diamides, nine typical backbone structures are expected to occur on their potential energy surface of *E*=*f*(*φ*, *ψ*), whereas, for β‐amino acid diamides, a similar *E*=*f*(*φ*, *θ*, *ψ*) hypersurface could have as many as 27 (=3×9) different backbone types. For cyclic amino acid, such as ACPC, the reduced conformational surface *E*=*f*(*φ*, *θ*≈60°, *ψ*) comprises only 18 (=2×9) minima.^4^, ^24^, ^25^ Our main objective is to present a robust approach to assign characteristic ECD spectra to secondary structures from the appropriate VCD spectrum, which is straightforward to calculate by using an appropriate DFT method. The experimental ECD^exp^(*λ*) assignment will complete the protocol outlined below (Scheme 3):

1. Both ECD^exp^(*λ*) and VCD^exp^(*ṽ*) of the oligomer (foldamer) are measured.
2. The conformationally relevant structures (minimum‐energy structures) of the oligomer are obtained both by MDCA and Monte Carlo multiple minimum (MCMM) conformer searches.
3. The topologically different, low‐energy conformers are geometry‐optimized by using a suitable DFT method: both conformers and their relative weights (*c_i_*) are determined.
4. For each of the conformers, the VCD spectrum (VCD_*i*_^calc^(*ṽ*)) is calculated (e.g., at the B3LYP/6‐311+G(d,p) level of theory).
5. VCD^exp^(*ṽ*) is compared with the sum of the population‐weighted individual spectra: VCD^calc^(*ṽ*)=Σ*c_i_*VCD_*i*_^calc^(*ṽ*).
6. The semiquantitative similarity of VCD^exp^(*ṽ*) to **Σ** *c_i_*VCD_*i*_^calc^(*ṽ*) validates both the conformational weights (*c_i_*) and the calculated conformers.
7. The validated ensemble is used to calculate the ECD_*i*_^calc^(*λ*) spectrum (at a given level of theory, for example, B3LYP/6‐311+G(d,p)) as Σ*c*_***i***_ECD_*i*_^calc^(*λ*).
8. The (semi)quantitative similarity between Σ*c_i_*ECD_*i*_^calc^(*λ*) and ECD^exp^(*λ*) ensures the structural assignment of the measured ECD spectrum.

Unlike ECD, which is limited to a few amide chromophore transitions (e.g., n→π*, π→π*), in the case of VCD, the chemical bonds themselves are the chromophores; thus the spectra contain numerous vibrational bands that can be analyzed for each molecular arrangement. Thus, if the absolute configuration of the molecule is known, then the VCD technique is excellent for determining the backbone torsion angles, which allows for structural classification of the polyamide chain. Today, ab initio level VCD spectral calculations are more advanced than those targeting ECD.^26^ Thus, measuring and attempting to reproduce the VCD spectrum of a complex system is an excellent method to validate whether the correct conformational distribution has been obtained for a target system; in other words, to ensure that all relevant conformers are found and incorporated into the ensemble. These structures then can be used to calculate and analyze ECD spectra.^27^

Our interpreted example comprises a pair of furanuronic acid diamides (**1 a** and **2 a**) and their N‐methylated variants (**1 b** and **2 b**). The AFU building blocks are water‐soluble variants of ACPC, which is one of the most studied foldamer building blocks. Inserting an oxygen into the completely hydrophobic cyclopentyl side chain of ACPC affords β‐aminotetrahydrofuran carboxylic acid (ATFC). The −CH_2_−→O atom replacement eliminates steric repulsion between −CH_2_− and the amide NH and forms a favorable electrostatic interaction (O⋅⋅⋅HN) in the form of hydrogen‐bond‐stabilizing selected backbone conformers. This hydrogen bond in ATFC fixes and orients the C‐terminal amide plane, as measured by *ψ*, and thus, effectively reduces the available conformational space of the cyclic β‐amino acid residue.^25^, ^28^, ^29^, ^30^, ^31^, ^32^ (Such a hydrogen bond was identified in a close derivative based on α‐d‐xylofuranuronamide by X‐ray crystallography.^33^) MCMM conformational searches resulted in thousands of conformers, on average, the energetic ordering and population distribution of which was determined by DFT calculations. The most significant conformers are discussed below and grouped according to their basic structural features.

### Hydrogen‐bond‐stabilized conformers

**Figure 1:**
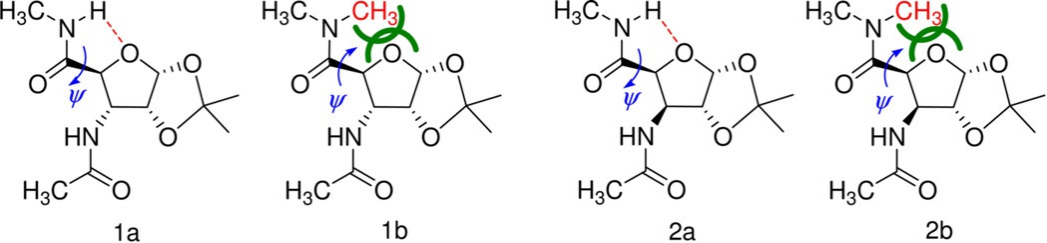
The four β‐sugar amino acid diamide foldamer building units studied herein. Angle *ψ* is restricted by the favorable intramolecular hydrogen bond in both **1 a** and **2 a** to a value of about +110°. Elimination of the hydrogen bond and introduction of steric repulsion (green lines), as in **1 b** and **2 b**, shifts the backbone structure markedly, and thus, *ψ*≈−120°.

**Figure 2:**
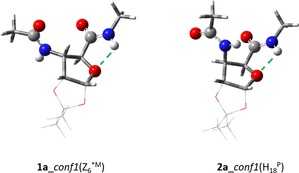
The two most abundant conformers of **1 a** and **2 a**: both have an intramolecular hydrogen bond acting as a conformer selector.

In building blocks **1 a** and **2 a** (Figure 1), the NH⋅⋅⋅O‐type intraresidual hydrogen bond is “active and operative”, and thus, poses a restriction on free rotation about torsion angle *ψ*. The strength of such conformational restriction is proportional to the strength of the hydrogen bond, which can be described by the donor–acceptor distance (*d*_N⋅⋅⋅O_ (Å) and *d*_H⋅⋅⋅O_ (Å)) and N−H⋅⋅⋅O angle (∡_N−H⋅⋅⋅O_ (°)). The closer the value of *d* is to about 2.4 Å and the angle of N−H⋅⋅⋅O is to about 120°, the stronger the hydrogen bond is and the more severe the restriction is. Conformations **1 a**_*conf1*, **2 a**_*conf1*, and **2 a**_*conf3* have short values of *d* (<2.7 Å) and the most optimal angle values (102.2 and 114.7°; Table 1 and Figure 2), which indicates that conformational selection occurs both for the ribo (Rib) and xylo (Xyl) configuration. In line with the above‐described assignment protocol, these rotamers are the most relevant backbone conformers both for **1 a** and **2 a** (Table 1 and Scheme 3, steps 2 and 3) because the relative conformational free energy of *conf1* is lowered by more than 9 kJ mol^−1^, compared with those of *conf2* for both **1 a** and **2 a**.

**Table 1:** Selected structural parameters of **1 a** and **2 a** with their configurations calculated at the B3LYP/6‐311+G(d,p) level of theory.

Model β ‐amino acid	*c_i_* ^[a]^ [%]	Δ *G* [kJ mol^−1^]	ϕ,θ,ψ [°]	*d*_N⋅⋅⋅O_ [Å]	α_NHO_ [°]	*d*_H⋅⋅⋅O_ [Å]	Assigned conformational code^[b]^
**1 a**							
*conf1*	98.2	0.0	−131.7, 83.1, 102.2	2.6	106.6	2.2	Z_6_^*M^
*conf2*	1.8	9.92	−129.6, 83.0, −121.7	3.4	72.7	3.6	H_18_^M^
**2 a**							
*conf1*	95.8	0.0	134.6, −40.7, 114.7	2.7	108.0	2.2	H_18_^P^
*conf2*	2.2	9.39	144.2, −41.0, −95.0	3.6	69.5	3.8	Z_6_^*P^
*conf3*	2.0	9.57	−75.0, −24.4, 115.4	2.6	107.5	2.2	Z_8_^P^

[a] Boltzmann distribution at *T*=298 K. [b] According to Beke et al.^4^

Based on the determined *(ϕ*, *θ*, *ψ)* dihedral triplets, compound **1 a** adopts a zigzag (Z_6_^*M^) conformation, whereas **2 a** has a helical (H_18_^P^) backbone structure.

VCD spectra of **1 a** and **2 a** were measured in [D_3_]MeCN, and the most informative C=O stretching motion of the amide group was analyzed and discussed in detail (Scheme 3, step 1). For all relevant conformers, *c_i_*>1 % (Table 1), the associated VCD spectra (VCD_*i*_^calc^(*ṽ*)) were calculated at the B3LYP/6‐311+G(d,p) level of theory (Scheme 3, step 4).

**Figure 3:**
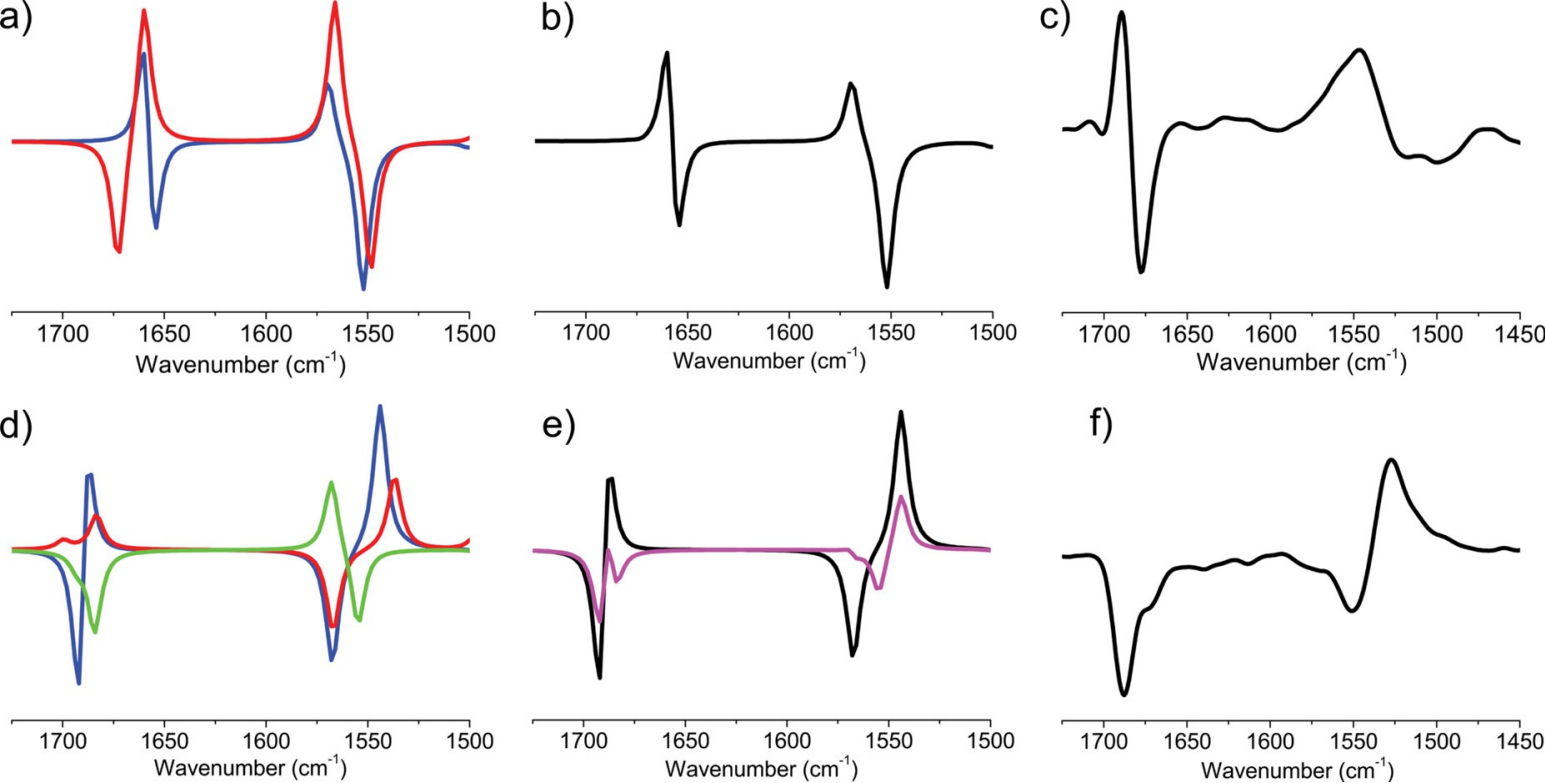
The pure VCD spectra of the conformers, VCD_i_^calc^(ṽ), calculated for **1 a** (a) and **2 a** (d); blue: *conf1*, red: *conf2*, green: *conf3*). b and e) Summation spectra (Σ*c_i_*VCD_*i*_^calc^(ṽ)) obtained from the calculated weights (black curves; see Table 1) and manually adjusted weights of **2 a**_*conf1* of 40 % and of **2 a**_*conf3* of 60 % (magenta curve). The experimental VCD^exp^(ṽ) spectra of **1 a** (c) and **2 a** (f).

The VCD^exp^(*ṽ*) of **1 a** shows a high resemblance to that of ΣVCD_*i*_^calc^(*ṽ*), and therefore, we conclude that **1 a** adopts predominantly a zigzag (Z_6_^*M^) backbone conformer in MeCN. The VCD^exp^(*ṽ*) of **2 a** has two negative amide I absorptions (*ṽ*=1688 and 1671 cm^−1^ (sh); Figure 3 f). However, Σ*c_i_*VCD_*i*_^calc^(*ṽ*) (Figure 3 e, black curve) shows poorer agreement with the experimental data, especially near *ṽ*=1685 cm^−1^. The calculated pure spectrum of **2 a**_*conf3* has a negative band at *ṽ*=1684 cm^−1^ (green curve, Figure 3 d), which is associated with a backbone conformer that has a hydrogen bond between the C‐terminal amide NH and the ring O atom. The latter conformer differs from that of **2 a**_*conf1* in terms of its *φ* value (+134.6°, relative to −75.0°; Table 1); thus **2 a**_*conf1* and **2 a**_*conf3* are somewhat similar. Varying the weights of the pure components (in a manual, reverse principal component analysis (PCA)‐like iteration) to more closely reproduce the measured spectrum, we established corrected contribution values of **2 a**_*conf1* of 40 % and of **2 a**_*conf3* of 60 % (Figure 3 e, magenta). Because these values give a better agreement with the experimentally determined spectrum, we may conclude that the structure of **2 a** in MeCN is a mixture of H_18_^P^ and Z_8_^P^ conformers.^4^, ^25^

**Figure 4:**
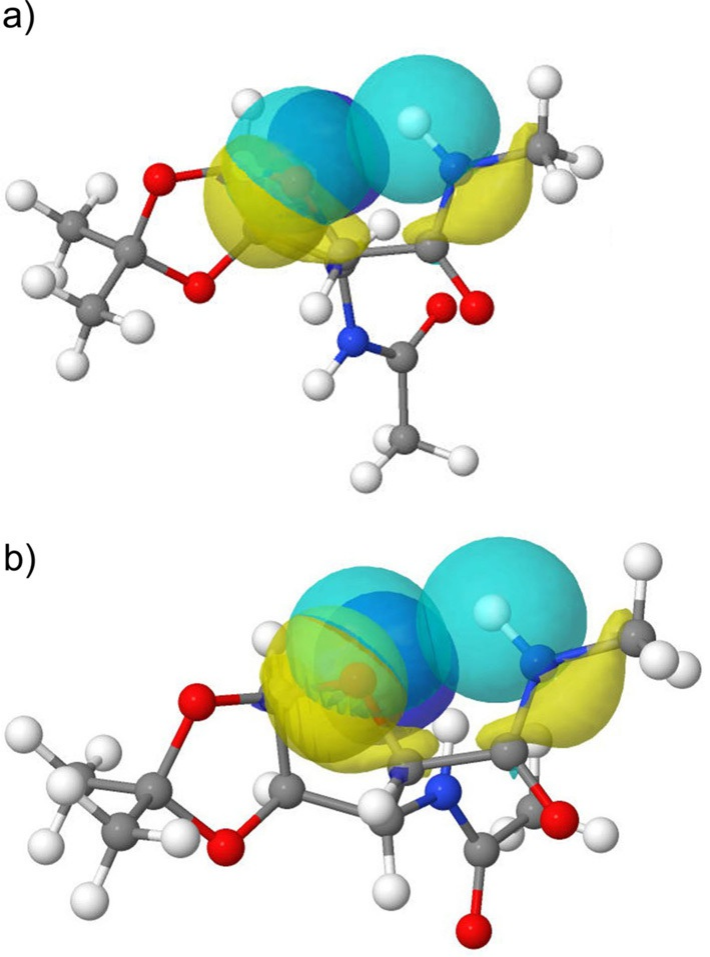
Scaffold of two selected conformers of **1 a** and **2 a**, with selected natural bond orbitals (NBOs) highlighting the intraresidual hydrogen bonds: a) **1 a**_*conf1* and b) **2 a**_*conf1*. Pale blue on the right: NH bond, yellow: NH lone pair, pale blue on the left: O_ring_ lone pair, dark blue: O_ring_ lone pair.

The intraresidual hydrogen bond exists in all relevant conformers (Figure 4), forming a five‐membered pseudo‐ring; this motif is also found in several structures deposited in the Cambridge Structural Database.^34^ This structure‐stabilizing interaction influences both the molecular scaffold and the macroscopically observed physicochemical properties (e.g., solubility, lipophilicity, and permeability), which are important aspects of drug design and discovery.^35^ Similar intramolecular hydrogen bonds were described by Saludes et al. in a sialic acid (SAA) foldamer, by Sharma et al. in the case of other SAAs, and in a foldamer made from a phenylisoserine derivative.^28^, ^29^, ^30^, ^36^

### Conformers shifted by steric repulsion

If the intramolecular hydrogen bond is “turned off” and, at the same time, considerable steric repulsion is introduced by an additional methyl group (H→CH_3_; Figure 1) then a significant conformational shift of *ψ* is seen both for **1 b** and **2 b**. Therefore, both the conformer type and population, and thus, the VCD and ECD spectra of these models, should be markedly different from those of **1 a** and **2 a**. Continuing a systematic conformational space search (Scheme 3, step 2) and subsequent structure optimization (Scheme 3, step 3), the dominant backbone structures were once again elucidated for **1 b** and **2 b** (Table 2).

**Table 2:** Selected structural parameters of the **1 b** and **2 b** configurations.

Model β ‐amino acid	*c_i_* ^[a]^ [%])	Δ *G* [kJ mol^−1^]	ϕ,θ,ψ [°]	*d*_N⋅⋅⋅O_ [Å]	α_NHO_ [°]	*d*_H⋅⋅⋅O_ [Å]	Assigned conformational code^[b]^
**1 b**							
*conf1*	88.8	0.0	−142.5, 82.7, −161.2	3.1	70.3	3.3	H_14_^M^
*conf2*	6.1	6.64	−134.5, 73.9, 79.0	3.0	75.4	3.0	Z_6_^*M^
*conf3*	5.1	7.09	−110.0, 83.1, −109.9	3.5	58.1	4.1	H_12_^P^
**2 b**							
*conf1*	99.9	0.0	141.8, −39.4, −80.6	3.7	54.0	4.3	Z_6_^*P^
*conf2*	0.1	16.7	104.9, 41.7, −160.9	3.2	69.2	3.4	Z_6_^M^

[a] Boltzmann distribution at *T*=298 K. [b] According to Beke et al.^4^.

**Figure 5:**
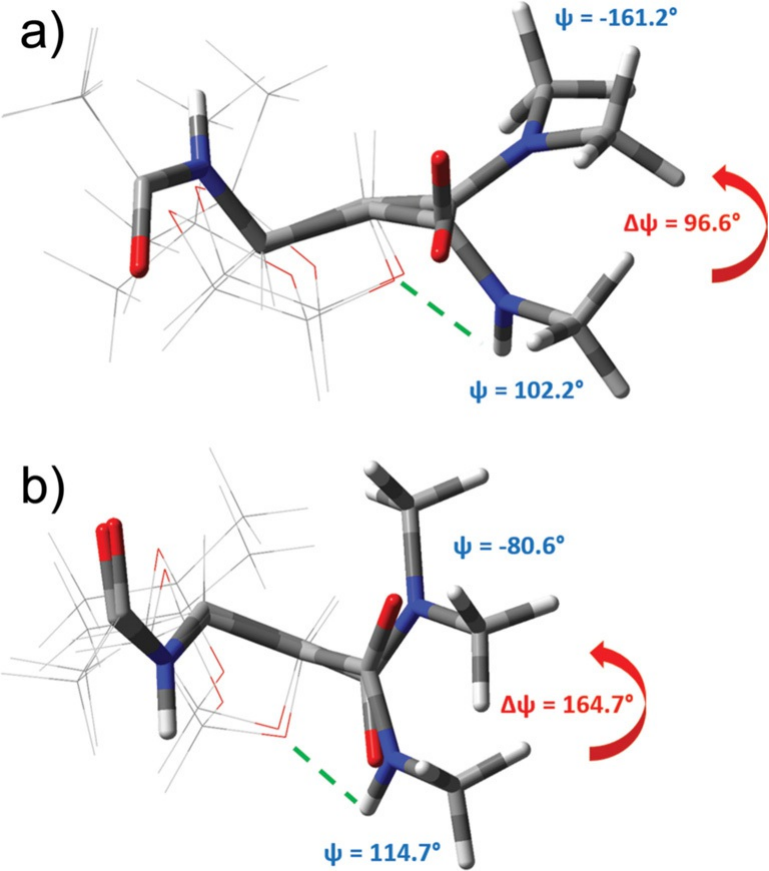
The superimposed major conformers (Table 2) of a) **1 a** on **1 b** and b) **2 a** on **2 b**. Green dashed lines indicate the hydrogen bond (turned‐on state) in the case of **1 a** and **2 a**; the red arrows show the conformational shift introduced by turning the hydrogen bond off and switching steric repulsion on (**1 b**, **2 b**).

We found that in both cases a strong conformer selection was operative: a single (*c_i_*>99 %), or at least a dominant (*c_i_*>89 %), backbone conformer was calculated for both stereoisomers. In the *conf1* structures of both **1 b** and **2 b**, the N⋅⋅⋅O_ring_ distance is remote. This steric hindrance reorients the C terminus of the building blocks, that is, *ψ* changes from +102.2° to −161.2° (Δ=96.6°) for **1 b**_*conf1* and from +114.7 to −80.6° (Δ=164.7°) for **2 b**_*conf1* (Figure 5). As a conclusion, **1 b** has a helical (H_14_^M^), while **2 b** a zigzag (Z_6_^*P^) backbone structure.^4^

**Figure 6:**
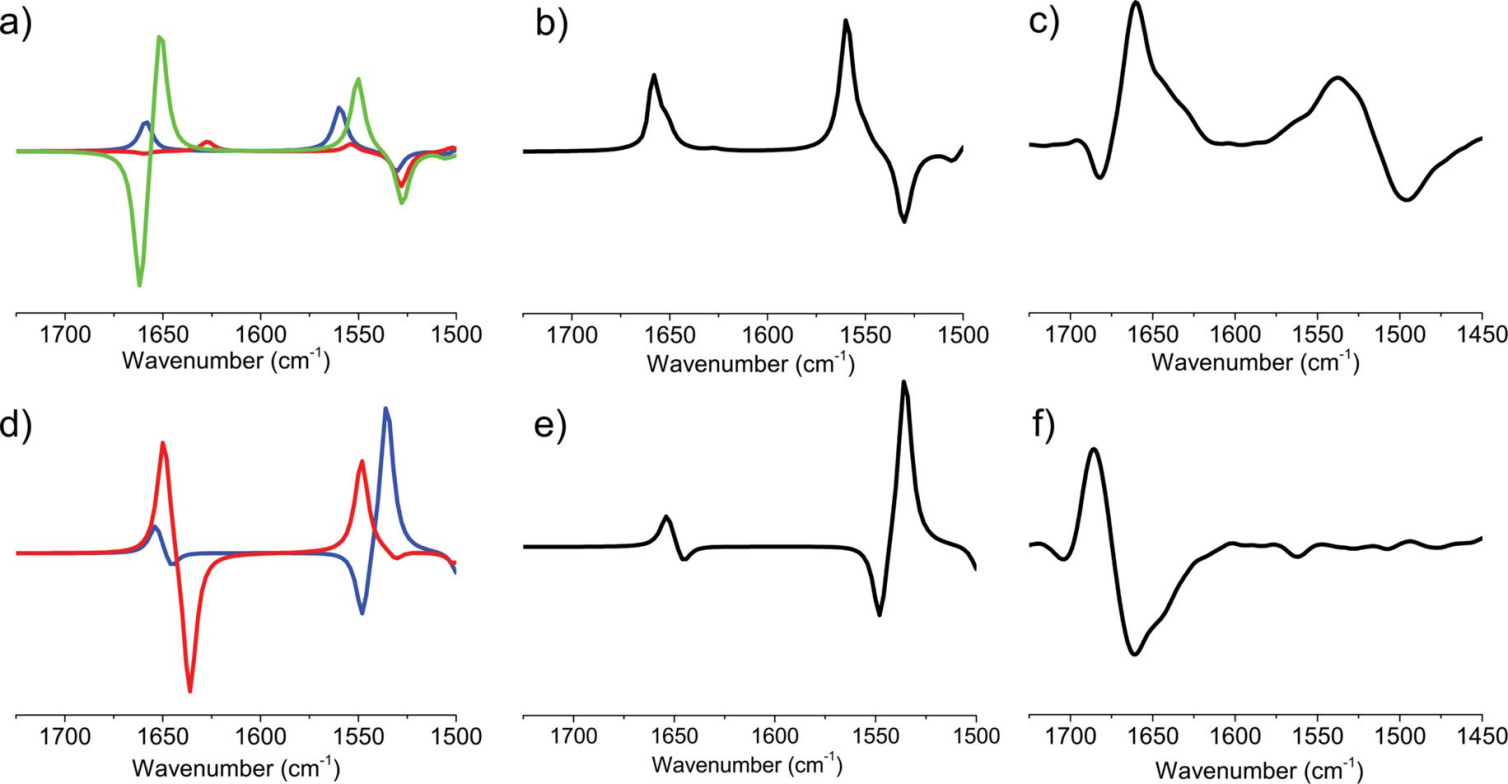
The pure VCD spectra of the conformers, VCD_*i*_^calc^(ṽ), of **1 b** (a) and **2 b** (d); blue: *conf1*, red: *conf2*; and green: *conf3*. Summation spectra (Σ*c_i_*VCD_*i*_^calc^(*ṽ*)) of **1 b** (b) and **2 b** (e) obtained from the calculated weights (see Table 2). Experimental VCD^exp^(*ṽ*) spectra of **1 b** (e) and **2 b** (f).

The experimental spectrum, VCD^exp^(*ṽ*), of *cis*‐**2 b** (Figure 6 f) has a “negative twist” in its amide I region, namely, a negative band (*ṽ* ≈1660 cm^−1^) is followed by positive VCD band (*ṽ* ≈1685 cm^−1^). On the other hand, *trans*‐**1 b** has a positive band (*ṽ* ≈1660 cm^−1^) with a distinct shoulder (*ṽ* ≈1640 cm^−1^; Figure 6 c). The VCD spectra calculated for the dominant conformers, VCD_*i*_^calc^(ṽ), of **1 b** and **2 b** (Table 2) show significant similarity to that of their experimental counterparts. However, if fine‐tuned with the minor forms, then **Σ** *c_i_*VCD_*i*_^calc^(*ṽ*) shows even better agreement with the experimental VCD spectra of both **1 b** and **2 b**. (Figure 6).

**Figure 7:**
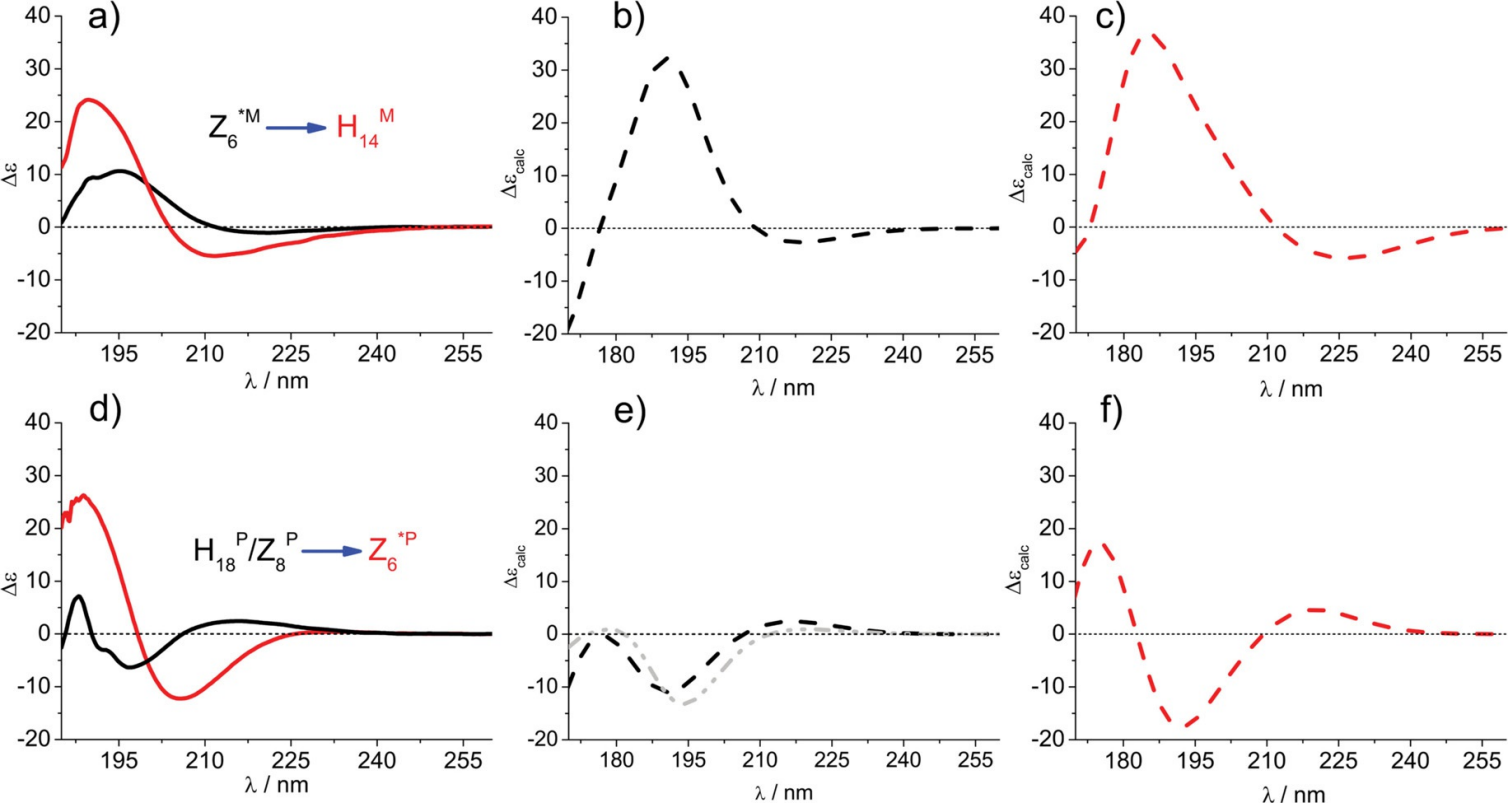
ECD^exp^(*λ*) *of* a) **1 a** (black) and **1 b** (red) and d) **2 a** (black) and **2 b** (red) recorded in MeOH. Reconstructed ECD spectra (Σ*c_i_*ECD_*i*_^calc^(*λ*)) obtained from the calculated weights (see Tables 1 and 2) for **1 a** (b), **1 b** (c), **2 a** (e), and **2 b** (f). The gray spectrum in e) was derived by using 0.4 of **2 a**_*conf1*+0.6 of **2 a**_*conf3*; that in f) was derived from 0.999 of **2 b**_*conf1*+0.001 of **2 b**_*conf2*.

The last and perhaps the most important question that remains is whether this approach can also be used to assign ECD spectra. In other words, is the ECD spectral change measured between **1 a** (Figure 7 a, black) and **1 b** (Figure 7 a, red) indeed associated with the backbone conformational change from Z_6_^*M^ to H_14_^M^? Similarly, the question is whether the difference measured between **2 a** (black) and **2 b** arises from the H_18_^P^/Z_8_^P^ to Z_6_^*P^ backbone amide group reorientation. The subtle difference in building block constitution (−H→−CH_3_) induces a net ECD spectral change, which manifests in terms of a distinct bathochromic shift for Rib derivatives (**1 a**→**1 b**), whereas for **2 a**→**2 b** a large difference in shape and intensity (*cis*, [3*R*,4*S*]) is measured (Figure 7).

The spectral similarity between ECD^exp^(*λ*) and Σ*c_i_*ECD_*i*_^calc^(*λ*) (Figure 7) is satisfactory, especially if we consider all existing theoretical challenges associated with ab initio ECD spectral calculations. For ECD, we need to compute the ground and excited states of each electronic transition to successfully determine their transition energies. The widely used method is time‐dependent (TD) DFT, in combination with the use of a higher level basis set (e.g., 6‐311+G(d,p)), since the calculation of the excited state is more sensitive to basis set issues than that of the ground state.^37^ The wise combination of two chiroptical methods (ECD‐ORD, ECD‐VCD; ORD=optical rotatory dispersion) may solve this problem because it seems that conformer types, relative weights, and spectral properties (VCD and ECD) are calculated at an acceptable similarity.^38^, ^39^, ^40^, ^41^

## Conclusion

**Figure 8:**
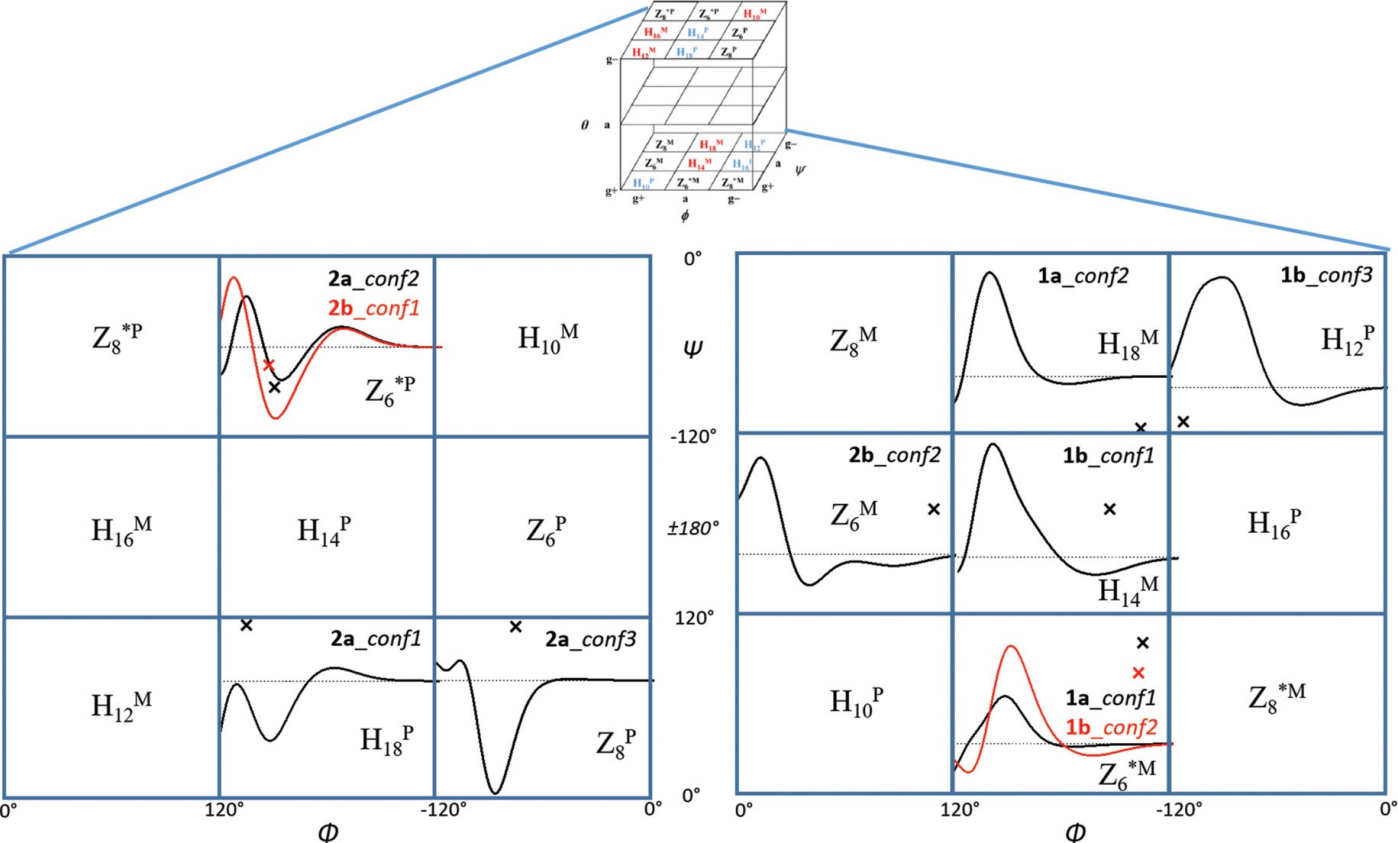
All possible backbone conformers of the current foldamer building blocks, **1 a**, **1 b**, **2 a**, and **2 b**, along with their pure ECD spectra, as calculated at the B3LYP/6‐311+G(d,p) level of theory, ECD_i_^calc^(*λ*). Red and black crosses indicate the location of a conformer on the (*φ*, *ψ*) subspace of the conformational hypercube (*φ*, *θ*, *ψ*).

We showed that it was possible to describe the conformational state of flexible building blocks (e.g., for β‐amino acid residues) in terms of major backbone conformers by selecting key structures from MDCA and MCMM refined by ab initio conformer optimization (e.g., B3LYP/6‐311+G(d,p)). These conformers (*conf1*, *conf2*, *conf*n) and their relative weights (*c_i_*) were successfully cross‐validated by calculating the associated VCD spectra, VCD_*i*_^calc^(*ṽ*), for each of them, and comparing their weighted sums, Σ*c_i_*VCD_*i*_^calc^(*ṽ*), to their experimental counterparts, VCD^exp^(*ṽ*). We found that replacing the attractive hydrogen bond with steric hindrance shifted the second amide plane, the *ψ* torsion, which was successfully monitored both by VCD and ECD spectral changes. The above conformational shift, expressed in terms of changes in conformer type (Z_6_^*M^→H_14_^M^ and H_18_^P^/Z_8_^P^→Z_6_^*P^), could be assigned to the ECD spectral variation observed. In this way, we assigned the pure ECD spectra, at least semiquantitatively, to all different backbone conformers. The associated pure ECD spectrum was determined for eight different backbone structures, namely, for four that were zigzag (Z_8_^P^, Z_6_^M^, Z_6_^*M^, Z_6_^*P^) and four helical (H_14_^M^, H_18_^P^, H_18_^M^, H_12_^P^; Figure 8).

One of the most basic concepts of foldamer chemistry is that, by simply controlling the stereochemistry of the building blocks, oligomers of designed, well‐defined, and stable secondary structure can be built.^42^ However, ECD properties of the building blocks are neither that strict nor that simple because any change in the amide plane orientation during secondary structure construction might shift or reshape the spectrum. Our results demonstrate that such changes are to be expected if monomers start to form oligomers and, for example, intramolecular hydrogen bonds are exchanged for more ideally oriented intermonomer interactions, similarly to the effect of N‐methylation in our example, and might lead to the evolution of unexpected secondary structural changes. Thus, the question of how sensitive ECD spectroscopy is to backbone changes of polyamides holds and can be answered. The present models show that it is impossible to predict the spectrum based solely on the absolute configuration of the monomeric unit. However, different amide plane orientations coupled to spectral changes, either of modest (Z_6_^*M^→H_14_^M^) or large scale (H_18_^P^/Z_8_^P^→Z_6_^*P^) (Figure 7 a and d), can be nicely tracked by means of ECD spectroscopy. By using the currently introduced method, it is possible to complete secondary structure assignment of foldamers, chimera, and so forth composed of common (α‐) and/or non‐proteinogenic amino acid residues of a given length.

## Experimental Section

### Computational details

Conformational mapping was performed by using MCMM searches with the OPLS3 force field,^43^ as implemented in the Schrödinger Suite (MacroModel Schrödinger LLC, New York, NY, 2019). 100 000 steps were carried out, involving the random variation (within the range of 0–180°) of a randomly selected subset of all rotatable torsional angles of the system. The perturbed structures were energy‐minimized and the unique structures were stored within a 21 kJ mol^−1^ energy window above the global minimum. The solvent effect was modeled by the GB/SA algorithm (with water as the solvent). The obtained structures were then optimized through the DFT method at the B3LYP/6‐311G+(d,p) level (Gaussian 09 software).^44^

Calculations of ECD spectra were completed by using the TD‐DFT method at the B3LYP/6‐311G+(d,p) level of theory, combined with the integral equation formalism polarizable continuum model (IEF‐PCM) solvent model for methanol. To simulate VCD spectra, harmonic vibrational frequencies and rotational strengths were calculated at the B3LYP/6‐311G+(d,p) level of theory, combined with the IEF‐PCM solvent model for acetonitrile as well. Although continuum solvation was not the ideal methodology for reproducing VCD spectra, ^[45]^ if results were treated with care, it was shown to be a rational alternative to explicit solvation in systems similar to ours.^46^

In the present calculations, DFT optimization preserved the force field derived conformations (with a root mean square deviation (RMSD) of (0.4±0.2) Å for non‐hydrogen atoms, similar to that reported previously^47^); however, if this was not the case, techniques such as normal‐mode optimization could be used to maintain the topology of conformers, while optimizing bond lengths and angles.^48^

To simulate ECD and VCD spectra, results were obtained by summing the calculated spectra of individual conformers multiplied by their calculated populations at room temperature based on the Boltzmann distribution (Tables 1 and 2).

### Spectroscopic measurements

The ECD spectra were recorded on a Jasco J‐810 spectropolarimeter at room temperature in a 0.02 cm quartz cell. The sample concentration was 1 mg mL^−1^; methanol (Uvasol, Merck, Darmstadt, Germany) was used as a solvent. All spectra were recorded between *λ*=185 and 260 nm. The ECD scans were performed 5 times with a scanning speed of 50 nm min^−1^. The resulting spectra were background corrected and smoothed by using the Means‐Movement algorithm. The ECD values were given in Δ*ϵ* (dm^3^ mol^−1^ cm^−1^).

All VCD spectra were recorded in [D_3_]MeCN with a Bruker PMA 37 VCD/PM‐IRRAS module connected to an Equinox 55 FTIR spectrometer at a resolution of 4 cm^−1^. The sample concentration was 10 mg mL^−1^. The ZnSe photoelastic modulator of the instrument was set to $\tilde{\nu }$ =1600 cm^−1^, and an optical filter with a transmission range of $\tilde{\nu }$ =1960–1250 cm^−1^ was used to increase the sensitivity in the amide spectral region. The instrument was calibrated for VCD intensity with a CdS multiple‐wave plate. A CaF_2_ cell of 0.2 mm path length was used. To improve the signal to noise (S/N) ratio, spectra were averaged for 5 h (corresponding to ca. 17 500 accumulated interferograms). Baseline correction was achieved by subtracting the spectrum of the solvent obtained under the same conditions.

### Synthesis of 1 a, 1 b, 2 a, and 2 b

#### 1,2:5,6‐Di‐O‐isopropylidene‐α‐d‐allofuranose

Methyl sulfoxide (24.51 mL, 345.75 mmol) dissolved in dry CH_2_Cl_2_ (60 mL) was added dropwise to a stirred solution of oxalyl chloride (15 mL, 171.51 mmol) in dry CH_2_Cl_2_ (210 mL) at −80 °C. The reaction mixture was stirred for an additional 15 min, and 1,2:5,6‐di‐*O*‐isopropylidene‐α‐d‐glucofuranose (15 g, 57.6 mmol) dissolved in dry CH_2_Cl_2_ (450 mL) was added dropwise. The mixture was allowed to react for 90 min at −80 °C and then triethylamine (TEA; 60.6 mL, 433.8 mmol) was added dropwise, maintaining the temperature at −70 °C. Finally, the reaction was quenched by adding NaBH_4_ (4.35 g, 116.25 mmol) dissolved in a mixture of EtOH/H_2_O (4:1, 300 mL). The temperature of the reaction mixture should be kept within −60 to −40 °C. The reaction mixture was extracted with CH_2_Cl_2_ (3×100 mL), dried over Na_2_SO_4_, and concentrated in vacuum. The crude product was recrystallized from cyclohexane to give a white solid (12 g, 80 %). M.p. 74–76 °C (Sigma Aldrich 73–76 °C).

### Final steps to produce diamide models

#### N,N‐Dimethyl‐1,2‐O‐isopropylidene‐3‐azido‐3‐deoxy‐α‐d‐ribofuranuronamide (1 a)

1,2‐*O*‐Isopropylidene‐3‐azido‐3‐deoxy‐α‐d‐ribofuranuronic acid (0.337 g, 1.47 mmol) was dissolved in dry THF (5.25 mL) and cooled at −15 °C. TEA (270 μL) and isobutyl chloroformate (250 μL) were added dropwise to the solution and stirred. The temperature was kept between −10 and −15 °C (solution I). Dimethylamine in THF (2 m, 5.5 mL) containing TEA (270 μL) was cooled at 0 °C (solution II) and added to solution I after 20 min. The reaction mixture was stirred for 1 h between −10 and 15 °C and 1 h at 0 °C, then the pH was checked (pH 7–8). The precipitate (TEA**⋅**HCl) was filtered and washed with cool THF. The solvent was removed; the residue was dissolved in EtOAc (20 mL); extracted with cool distilled water (2×10 mL); and washed with a solution of HCl (2 n, 2×10 mL), a saturated solution of NaHCO_3_ (2×10 mL), and brine (2×10 mL). The organic phase was dried (MgSO_4_) and concentrated in vacuum. The crude product was isolated and purified by column chromatography (eluent: hexane/EtOAc 1:2) to give colorless oil (0.164 g, 44 %). *R*_f_=0.57 (EtOAc/hexane 2:1); ^1^H NMR (CDCl_3_, 250 MHz): *δ*=5.76 (d, 1 H; H‐1), 4.66 (t, 1 H; H‐2), 4.53 (d, 1 H; H‐4), 4.17 (dd, 1 H; H‐3), 3.07 (s, 3 H; amide‐CH_3_), 2.94 (s, 3 H; amide‐CH_3_), 1.52 (s, 3 H; CH_3_), 1.30 ppm (s, 3 H; CH_3_); ESI‐MS: *m*/*z*: 257.3 [*M*+H].

#### N,N‐Dimethyl‐1,2‐O‐isopropylidene‐3‐azido‐3‐deoxy‐α‐d‐xylofuranuronamide (2 a)

From 1,2‐*O*‐isopropylidene‐3‐azido‐3‐deoxy‐α‐d‐xylofuranuronic acid (0.4 g, 1.75 mmol), the reaction was analogous to the reaction described for **1 a**. The product was a colorless oil (0.21 g, 48 %). *R*_f_=0.55 (EtOAc/hexane 2:1); ^1^H NMR (CDCl_3_, 250 MHz): *δ*=5.27 (d, 1 H; H‐1), 4.92 (d, 1 H; H‐4), 4.59 (d, 1 H; H‐2), 3.44 and 3.28 (s, 6 H; amide‐CH_3_), 3.2 (d, 1 H; H‐3), 1.8 and 1.63 ppm (s, 6 H; isopropylidene CH_3_); ESI‐MS: *m*/*z*: 257.2 [*M*+H].

#### N,N‐Dimethyl‐1,2‐O‐isopropylidene‐3‐amino‐3‐deoxy‐α‐d‐ribofuranuronamide

Compound **1 a** (0.201 g, 0.79 mmol) was dissolved in methanol (22 mL). The starting material was reduced with H_2_ on 10 % Pd/C by H‐Cube (parameters: 55 °C; 10 bar; 0.2 mL min^−1^). After the reaction, the solution was concentrated in vacuo to give the product as a colorless oil (0.157 g, 87 %). *R*_f_=0.74 (chloroform/methanol 3:1). The free amino group was detected with ninhydrin test and transformed without further analysis.

#### N,N‐Dimethyl‐1,2‐O‐isopropylidene‐3‐amino‐3‐deoxy‐α‐d‐xylofuranuronamide

From **2 a** (0.2 g, 0.79 mmol) the reaction was analogous to that of the reaction of **1 a**. The product was colorless oil (0.15 g, 85 %). *R*_f_=0.72 (chloroform/methanol 3:1). The free amino group was detected with ninhydrin test and transformed without further analysis.

#### N,N‐Dimethyl‐1,2‐O‐isopropylidene‐3‐acetamido‐3‐deoxy‐α‐d‐ribofuranuronamide (1 b)

*N*,*N*‐Dimethyl‐1,2‐*O*‐isopropylidene‐3‐amino‐3‐deoxy‐α‐d‐ribofuranuronamide (0.157 g, 0.68 mmol) was dissolved in a cooled mixture of pyridine (0.80 mL) and acetic anhydride (0.48 mL). The reaction mixture was allow to stand at 0 °C for 2 days. The solution was added dropwise into a mixture of water–ice (50 mL) and concentrated in vacuum. The crude product was recrystallized from ethyl acetate/petroleum ether to give a white solid (0.113 g, 61 %). *R*_f_=0.45 (ethyl acetate/methanol 4:1); ^1^H NMR (CDCl_3_, 700 MHz): *δ*=5.94 (d, 1 H; H‐1), 4.72 (br m, 2 H; H‐2, H‐3), 4.58 (d, 1 H; H‐4), 3.10 (s, 3 H; methylamide CH_3_), 2.97 (s, 3 H; amide‐CH_3_), 2.03 (s, 3 H; acetamide CH_3_), 1.58 (s, 3 H; isopropylidene CH_3_), 1.37 ppm (s, 3 H; isopropylidene CH_3_); ESI‐MS: *m*/*z*: 273.3 [*M*+H].

#### N,N‐Dimethyl‐1,2‐O‐isopropylidene‐3‐acetamido‐3‐deoxy‐α‐d‐xylofuranuronamide (2 b)

From *N*,*N*‐dimethyl‐1,2‐*O*‐isopropylidene‐3‐amino‐3‐deoxy‐α‐d‐xylofuranuronamide (0.15 g, 0.65 mmol), the reaction was analogue to that of **1 b**. The product was a white solid (0.177 g, 55 %). *R*_f_=0.43 (ethyl acetate/methanol 4:1); ^1^H NMR (CDCl_3_, 250 MHz): *δ*=5.97 (d, 1 H; H‐1), 4.96 (d, 1 H; H‐4), 4.61 (d, 1 H; H‐2), 4.5 (m, 1 H; H‐3), 3.62 (d, 1 H; H3), 2.96 and 2.81 (s, 6 H; amide‐CH_3_), 1.81 (s, 3 H; acetamide CH_3_), 1.4 (s, 3 H; isopropylidene CH_3_), 1.18 ppm (s, 3 H; isopropylidene CH_3_); ESI‐MS: *m*/*z*: 273.3 [*M*+H].

## Conflict of interest

The authors declare no conflict of interest.

## Supporting information

As a service to our authors and readers, this journal provides supporting information supplied by the authors. Such materials are peer reviewed and may be re‐organized for online delivery, but are not copy‐edited or typeset. Technical support issues arising from supporting information (other than missing files) should be addressed to the authors.

Supplementary
