# Supplementary material for: Assignment of Vibrational Circular Dichroism Cross‐Referenced Electronic Circular Dichroism Spectra of Flexible Foldamer Building Blocks: Towards Assigning Pure Chiroptical Properties of Foldamers
Source: Chemistry. 2019 Oct 23;25(65):14890–900. doi: 10.1002/chem.201903023 (PMC6899845; doi:10.1002/chem.201903023)

CHEMISTRY
A European Journal

Supporting Information

Assignment of Vibrational Circular Dichroism Cross-Referenced Electronic Circular Dichroism Spectra of Flexible Foldamer Building Blocks: Towards Assigning Pure Chiroptical Properties of Foldamers

Viktor Farkas,*[a] Adrienn Nagy,[b] Dóra K. Menyhárd,[a] and András Perczel*[a, b]

chem_201903023_sm_miscellaneous_information.pdf

Supporting Information

^{1}H NMR spectra

N-Methyl-1,2-*O*-isopropylidene-3-acetamido-3-deoxy-α-D-ribofuranuronamid (**1a**):

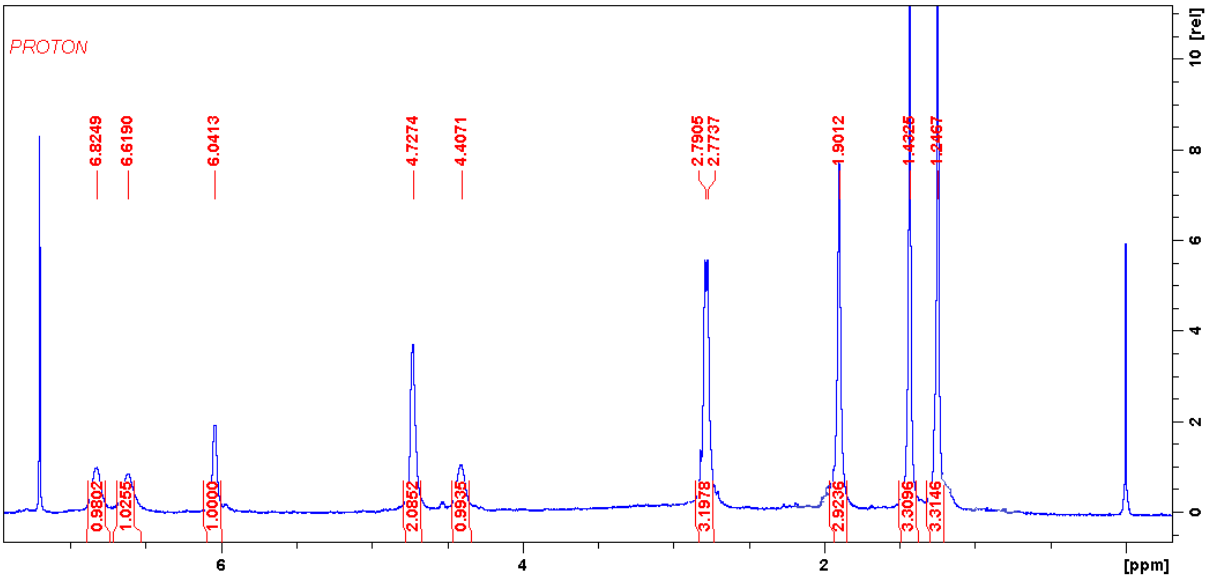

N-Methyl-1,2-*O*-isopropylidene-3-acetamido-3-deoxy-α-D-xylofuranuronamid (**2a**):

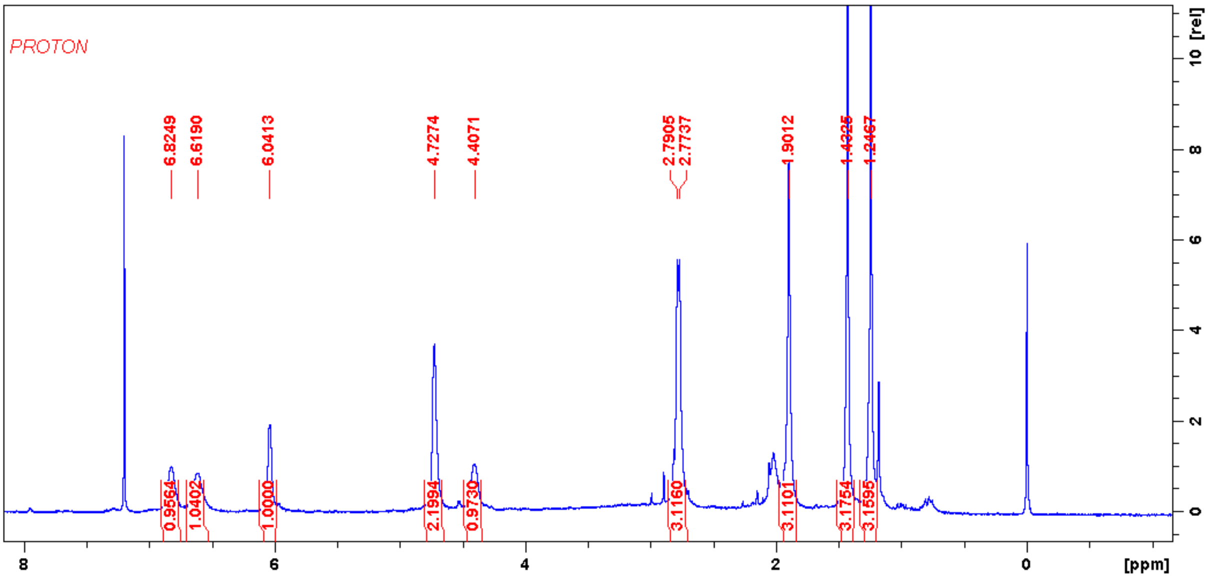

Supplement: Supplementary file 1 — Supplementary [file CHEM-25-14890-s001.pdf]
